# Bimekizumab, a Novel Humanized IgG1 Antibody That Neutralizes Both IL-17A and IL-17F

**DOI:** 10.3389/fimmu.2020.01894

**Published:** 2020-08-21

**Authors:** Ralph Adams, Asher Maroof, Terry Baker, Alastair D. G. Lawson, Ruth Oliver, Ross Paveley, Steve Rapecki, Stevan Shaw, Pavan Vajjah, Shauna West, Meryn Griffiths

**Affiliations:** ^1^Discovery Science, New Modality Therapeutics, UCB Pharma, Slough, United Kingdom; ^2^Immuno-Bone Therapeutic Area, Immunology Research, UCB Pharma, Slough, United Kingdom; ^3^Research Fellow, UCB Pharma, Slough, United Kingdom; ^4^Development Science, QP/DMPK, UCB Pharma, Slough, United Kingdom; ^5^Immuno-Bone Therapeutic Area, Immuno-Bone Discovery, UCB Pharma, Slough, United Kingdom; ^6^Translational Medicine, TM Immuno-Bone, UCB Pharma, Slough, United Kingdom

**Keywords:** anti-IL-17A, IL-17A, IL-17F, monoclonal antibody, bimekizumab, dual neutralization, dual targeting

## Abstract

Interleukin (IL)-17A is a key driver of inflammation and the principal target of anti-IL-17 therapeutic monoclonal antibodies. IL-17A, and its structurally similar family member IL-17F, have been shown to be functionally dysregulated in certain human immune-mediated inflammatory diseases such as psoriasis, psoriatic arthritis, and axial spondyloarthritis. Given the overlapping biology of these two cytokines, we postulated that dual neutralization of IL-17A and IL-17F may provide a greater depth of clinical response in IL-17-mediated diseases than IL-17A inhibition alone. We identified 496.g1, a humanized antibody with strong affinity for IL-17A but poor affinity for IL-17F. Affinity maturation of 496.g1 to 496.g3 greatly enhanced the affinity of the Fab fragment for IL-17F while retaining strong binding to IL-17A. As an IgG1, the affinity for IL-17A and IL-17F was 3.2 pM and 23 pM, respectively. Comparison of 496.g3 IgG1 with the commercially available anti-IL-17A monoclonal antibodies ixekizumab and secukinumab, by surface plasmon resonance and in a human *in vitro* IL-17A functional assay, showed that 496.g3 and ixekizumab display equivalent affinity for IL-17A, and that both antibodies are markedly more potent than secukinumab. In contrast to ixekizumab and secukinumab, 496.g3 exhibited the unique feature of also being able to neutralize the biological activity of IL-17F. Therefore, antibody 496.g3 was selected for clinical development for its ability to neutralize the biologic function of both IL-17A and IL-17F and was renamed bimekizumab (formerly UCB4940). Early clinical data in patients with psoriasis, in those with psoriatic arthritis, and from the Phase 2 studies in psoriasis, psoriatic arthritis, and ankylosing spondylitis, are encouraging and support the targeted approach of dual neutralization of IL-17A and IL-17F. Taken together, these findings provide the rationale for the continued clinical evaluation of bimekizumab in patients with immune-mediated inflammatory diseases.

## Introduction

Interleukin (IL)-17A was the first identified member of a family of six structurally similar cytokines; IL-17A, IL-17B, IL-17C, IL-17D, IL-17E (also known as IL-25), and IL-17F (1). Originally cloned in 1993 (2) as a cytokine derived from activated T cells, IL-17A is now recognized as a key pro-inflammatory cytokine in chronic immune-mediated inflammatory diseases, particularly psoriasis, and spondyloarthritis (3). The IL-17 axis has been shown to play an important role in defense against extracellular bacteria and fungi, through the induction of chemokines involved in the recruitment of neutrophils and monocytes. In a variety of murine disease models, IL-17A has also been demonstrated to have a crucial function in promoting chronic inflammation and autoimmunity (4–6). Among IL-17 family members, IL-17F is closest in sequence to IL-17A, sharing ~50% structural homology (7). Expressed as homodimers, or as a heterodimer (IL-17A/F) (8, 9), both IL-17A and IL-17F signal through the same heterodimeric complex of IL-17 receptors A and C (IL-17RA/RC) (10). The majority of non-hematopoietic cells have the potential to respond to the localized production of IL-17A or IL-17F as a result of the ubiquitous expression of their specific receptors.

Both IL-17A and IL-17F cytokines are expressed by Th17 cells, as well as additional immune cell types, including CD8 T cells, natural killer T cells, lymphoid tissue inducer cells, innate lymphoid cells and γδ T cells (11). Although common pathways are involved in the differentiation of Th17 cells, emerging data suggest that the regulation of IL-17A expression is distinct from that of IL-17F (12). IL-17A has a stronger affinity for the IL-17RA/RC complex, and thus promotes a greater induction of pro-inflammatory genes than IL-17F. Early mouse model data showed that animals lacking either IL-17A or IL-17F exhibited distinct biology (13). In contrast to IL-17A, IL-17F was thought to drive models of lung inflammation, with little or no role in experimental autoimmune encephalitis (14). However, human translational data showed that, like IL-17A, IL-17F synergizes with tumor necrosis factor (TNF) to induce a pro-inflammatory gene signature that is qualitatively similar to that induced by the combination of IL-17A and TNF (15, 16). Dysregulated expression of IL-17A and IL-17F is associated with chronic inflammatory diseases such as psoriasis, psoriatic arthritis, rheumatoid arthritis, ankylosing spondylitis, and asthma (17–20). Although IL-17A is known to be the more potent of the two cytokines, IL-17F is the more abundantly expressed of the two in psoriasis and spondyloarthritis (21). Despite the paucity of data in murine models supporting a role for IL-17F in promoting inflammation, human genetic data in individuals with autosomal dominant mutations in IL-17F, suggest a previously underestimated role for this cytokine (22).

Inhibiting IL-17A has proven to be an effective therapeutic strategy in the clinic. Two anti-IL-17A antibodies, ixekizumab, and secukinumab, are approved for the treatment of patients with psoriasis, psoriatic arthritis, and ankylosing spondylitis (23, 24). Furthermore, head-to-head trials demonstrated the superior clinical efficacy of ixekizumab and secukinumab in psoriasis over established treatments, ustekinumab (anti-IL-12/IL-23 monoclonal antibody) and etanercept (soluble TNF receptor inhibitor), respectively (25, 26).

Given, IL-17A and IL-17F share overlapping biology, we postulate that IL-17F also contributes to chronic tissue inflammation, beyond the established role of IL-17A. This rationale supports our hypothesis that neutralization of both IL-17A and IL-17F may be more effective than inhibition of IL-17A alone to neutralize IL-17-driven pathology.

In this study we describe the generation and characterization of 496.g3 (known as bimekizumab, formerly UCB4940), a humanized monoclonal antibody with high affinity for both IL-17A and IL-17F.

## Methods

### Preparation of Antibody Constructs and Expression

DNA encoding the light chain variable regions of 496.g1 and 496.g3 were cloned into UCB expression vectors containing DNA encoding human light chain Cκ. DNA encoding the shared heavy chain variable region of 496.g1 and 496.g3 was cloned into UCB expression vectors containing DNA encoding either human heavy chain γ1 C_H_1 region to generate Fab or human heavy chain γ1 IgG regions to generate IgG1. Antibodies were transiently expressed in CHO-S XE cells, a CHO-K1 derived cell line (27).

### Purification of Fab and IgG1

Fab and IgG proteins were purified from culture supernatants using affinity chromatography. Supernatants containing Fab were passed over a HiTrap Protein G column (GE Healthcare, Buckinghamshire, UK) and supernatants containing IgG were passed over a MabSelect™ SuRe™ column (GE Healthcare). Following a washing step with phosphate buffered saline (PBS) (pH 7.4), the bound material was eluted with 0.1 M glycine (pH 3.2) and neutralized with 2 m Tris-HCl (pH 8.5). Fractions containing Fab or IgG were pooled, quantified by absorbance at 280 nm, and concentrated using Amicon Ultra centrifugal filters (Merck Millipore, Massachusetts, USA). To isolate the monomeric fractions of Fab and IgG, we used size-exclusion chromatography over a HiLoad 16/60, Superdex 200 column (GE Healthcare) equilibrated with PBS (pH 7.4). Fractions containing monomeric Fab or IgG were pooled, quantified, concentrated, and stored at 4°C.

### Enzyme-Linked Immunosorbent Assay (ELISA)

Standard ELISA plates (Nunc Maxisorp^TM^, ThermoFischer Scientific, Massachusetts, USA) were coated with 1 μg/mL cytokine (IL-17A, IL-17B, IL-17C, IL-17D, IL-17E, and IL-17F) in PBS pH 7.4 overnight. Plates were washed three times in wash buffer (PBS supplemented with 0.05% Tween20, SigmaAldrich, Missouri, USA) and tapped dry. Diluted antibody was added to the relevant wells and incubated for 1 h at room temperature. Plates were washed three times and a goat IgG-horseradish peroxidase conjugated antibody with specificity for human Fc (Jackson ImmunoResearch, Pennsylvania, USA) was added to each well. Plates were incubated for 1 h at room temperature. Plates were washed for a final time before the addition of TMB (3,3′,5,5′-tetramethylbenzidine) Stabilized Substrate (Promega, Southampton, UK) for 8 min, after which an equal volume of stop solution (1 M H_3_PO_4_) was added to each well. Absorbance was then measured at 450 nm using a plate spectrophotometer (Biotek Instruments).

### Expression and Purification of IL-17A and IL-17F for Neutralization Bioassay

IL-17F was cloned into an in-house mammalian expression vector and expressed by transient transfection using the Expi293™ Expression System (Life Technologies). IL-17F protein was purified by cation exchange, followed by isolation of the dimer fraction by size exclusion chromatography.

IL-17A was cloned into an in-house mammalian expression vector upstream of the human IgG1 Fc coding region with a TEV (tobacco etch virus) cleavage site. IL-17A-Fc protein was expressed transiently in CHO-S XE cells. IL-17A protein was purified by Protein A affinity chromatography before the human Fc tag was removed using a TEV protease (produced in-house). A fraction containing untagged dimeric IL-17A protein was then isolated by size exclusion chromatography.

### Neutralization Bioassay

The potency of antibody 496 variants, ixekizumab (Taltz® Eli Lilly, Indiana, USA) or secukinumab (Cosentyx® Novartis, Basel, Switzerland), for the neutralization of human IL-17A and IL-17F was determined using a human primary cell bioassay. Normal human dermal fibroblasts (NHDFs) derived from neonate foreskin (106-05n, Sigma-Aldrich, Missouri, USA) were cultured in Dulbecco's Modified Eagle's Medium supplemented with 10% of heat-inactivated low endotoxin fetal bovine serum and 2 mM L-glutamine (Invitrogen, California, USA). Cells were grown in T75 flasks until 80–90% confluent, before being removed using 0.25% Trypsin-EDTA and plated in 384 well-plates (Corning, New York, USA) at 1,250 cells per well. Cells were allowed to rest for 3 h before addition of cytokines and/or antibodies. IL-17A or IL-17F were incubated with TNF for 1 h prior to being added to the cells. IL-6 protein quantification was determined using homogeneous time resolved fluorescence (HTRF; Cisbio, Codolet, France), as per the manufacturer's instructions, using a recombinant IL-6 standard curve (R&D Systems, Minnesota, USA) detected at 18 h (+/– 2 h). IL-6 levels were plotted against inhibitor concentrations in Prism 6 (GraphPad, California, USA) to generate IC_50_ and IC_90_ values.

### Surface Plasmon Resonance (SPR)

The binding affinities and kinetic parameters for the interactions of antibodies were determined by SPR on a Biacore T200 using Series S CM5 sensor chips (GE Healthcare Bio-Sciences AB, Uppsala, Sweden). HBS-EP (10 mM HEPES [pH 7.4], 150 mM NaCl, 3 mM EDTA, 0.05% v/v surfactant P20) was used as running buffer. All experiments were performed at 25°C. The antibody samples were captured using F(ab′)_2_ fragment-specific or Fcγ-specific Affinipure F(ab′)_2_ fragment goat anti-human IgG (Jackson ImmunoResearch, Pennsylvania, USA). Covalent immobilization of the capturing antibody was achieved by standard amine coupling chemistry to a level of 3,500–5,000 response units (RU).

Human IL-17A and IL-17A/F, cynomolgus macaque IL-17A and IL-17F (all generated at UCB) and human IL-17F (R&D Systems) were titrated over the captured purified antibody from 10 nM (IL-17F) or 5 nM (IL-17A and IL-17A/F) to 0.625 nM or 0.315 nM, respectively. Each assay cycle consisted of first capturing the antibody sample using a 1-min injection at a flow rate of 10 μL/min, followed by an association phase consisting of a 3-min injection of the IL-17 cytokine at a flow rate of 30 μL/min; dissociation was then monitored. After each cycle, the capture surface was regenerated at a flow rate of 10 μL/min with a 1-min injection of 40 mM HCl followed by a 30-sec injection of 10 mM or 5 mM NaOH. A blank flow-cell was used for reference subtraction and buffer-blank injections were included to subtract instrument noise and drift. Kinetic parameters were determined using Biacore T200 Evaluation Software V3.0.

## Results

### Discovery and Characterization of 496.g1

**Figure 1:**
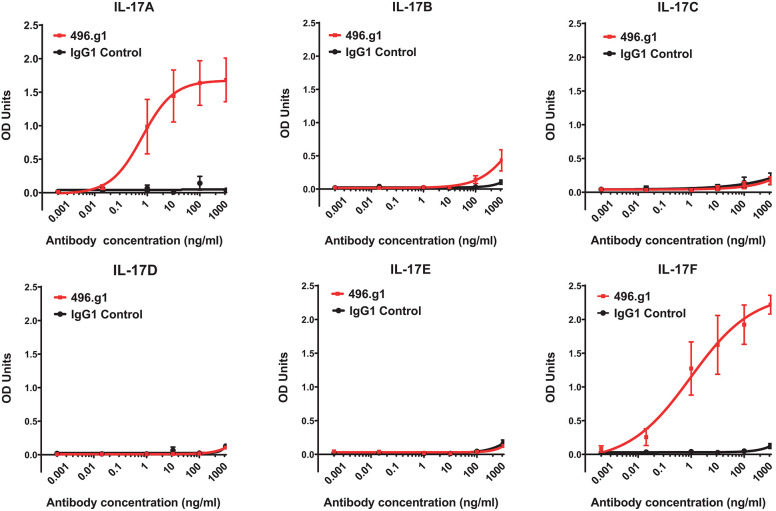
Binding of 496.g1 to human recombinant IL-17A–F. Recombinant cytokines **(A–F)** were coated onto high-binding ELISA plates. Titrations of 496.g1 or isotype control antibody were added, starting at concentrations of 10 μg/mL. Optical density (OD) absorbance was measured at 450 nm. Values represent average absorbance and standard deviation of six technical replicates.

Initially, we sought to generate an IL-17A therapeutic antibody. In brief, a panel of antibodies was raised in Sprague Dawley rats to human IL-17A. Using a single B cell selection method (28), the parental antibody 496 was identified for its strong binding to IL-17A and its ability to inhibit IL-17A-induced IL-6 production in the 3T3-NIH cell line (data not shown). Sequence alignment of IL-17 family members showed that IL-17B, IL-17C, IL-17D, IL-17E, and IL-17F shared 20–50% homology to IL-17A at the amino acid level. To determine whether the humanized variant of 496, 496.g1, was cross-reactive with other IL-17 family members, an indirect ELISA using recombinant protein was performed. Relative to an isotype-matched control antibody, 496.g1 showed binding to IL-17A (EC_90_ 12.1 ng/mL) and IL-17F (EC_90_ 358.5 ng/mL). Little or no binding to human IL-17B, IL-17C, IL-17D, and IL-7E was observed (Figure 1).

### Mathematical Modeling of IL-17A and IL-17F

**Figure 2:**
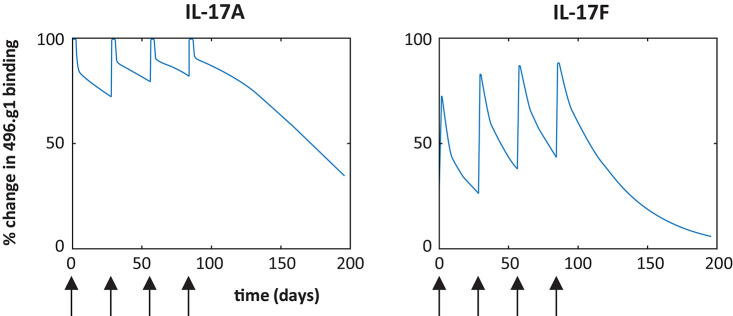
Predicted percentage of IL-17A and IL-17F bound to 496.g1 in psoriatic skin based on a target-mediated drug disposition model. Simulations are based on a 160 mg dose IV every 4 weeks (arrow) and partitioning of 30% of the antibody into the skin and indicate insufficient binding of 496.g1 to completely inhibit IL-17F in psoriatic skin.

To investigate the therapeutic potential of 496.g1, a target-mediated drug disposition model was used to predict the percentage of IL-17A or IL-17F bound to 496.g1 in skin (29). Allometric scaling, which considers the differences in body surface area and weight, was used to predict the pharmacokinetic parameters (clearance and volume of distribution) of 496.g1 in humans. In each case, the percentage of antibody that was ascribed to partition to the skin was 30% (30). The simulations predicted that, following a 160 mg IV dose of 496.g1 every 4 weeks, IL-17A was completely bound in plasma and >95% bound in skin compartments at trough or before the next dose was administered at steady state, but skin IL-17F showed <50% occupancy at the same timepoint (Figure 2). This was considered to be sub-optimal in humans as IL-17F signaling would not be completely inhibited. We had hypothesized that both IL-17A and IL-17F needed to be neutralized to attain optimal clinical outcomes compared with inhibition of IL-17A alone. Therefore, it was decided to try to improve the affinity of 496.g1 for IL-17F, while maintaining affinity for IL-17A.

### Characterization of 496.g3

**Figure 3:**
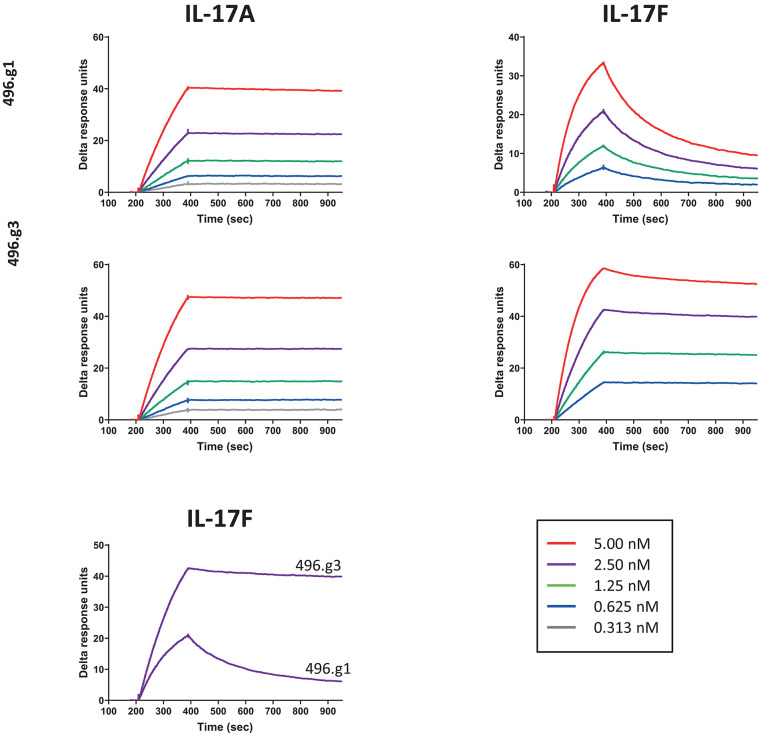
Affinity of 496.g3 to IL-17A and IL-17F. Anti-human F(ab′)_2_ was immobilized onto a CM5 sensor chip surface followed by the capture of either 496.g1 or 496.g3 Fabs. The association phase showed an increase in response over time following the injection of varying concentrations of IL-17A (5–0.313 nM) and IL-17F (5–0.625 nM) and this was followed by the dissociation phase when buffer replaced the IL-17A and IL-17F.

To affinity mature 496.g1 for IL-17F, a crystal structure of 496.g1 Fab in complex with IL-17F was generated and a proprietary *in silico* design method applied to the interface (details of which can be found in patent application number WO2014198951A2). Using this method, we tested a series of mutation combinations, identifying five mutations in the light chain variable region of 496.g1 that increased binding affinity for IL-17F while also improving affinity for IL-17A, giving rise to antibody 496.g3 (Supplementary Data). As a purified Fab fragment, the affinity constants (K_D_) of 496.g3 for IL-17F and IL-17A were shown to be 35 pM and 7 pM, respectively (Table 1). This compared favorably with the K_D_ of 496.g1 Fab for IL-17F and IL-17A at 1510 pM and 29 pM, respectively, showing a 43-fold increase in the affinity of 496.g3 Fab for IL-17F and a 4-fold increase in its affinity for IL-17A compared with 496.g1 (Figure 3).

**Table 1:** Binding affinities and kinetic parameters of 496.g1 and 496.g3 Fab fragments.

**Cytokine**	**496**	**k_**a**_ (M^**−1**^s^**−1**^)**	**k_**d**_ (s^**−1**^)**	**K_**D**_ (M)**	**K_**D**_ (pM)**
IL-17F	g1	4.00E+06	6.03E-03	1.51E-09	1,510
	g3	4.75E+06	1.64E-04	3.45E-11	35
IL-17A	g1	1.93E+06	5.63E-05	2.92E-11	29
	g3	1.44E+06	1.03E-05	7.20E-12	7

*Association (k_a_) and dissociation (k_d_) rates and affinity constants (K_D_) were determined by surface plasmon resonance*.

**Figure 4:**
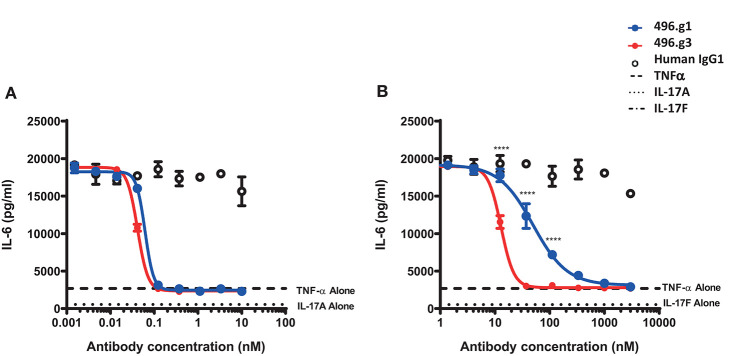
Inhibition of IL-6 production from normal human dermal fibroblasts (NHDFs) stimulated with TNF in combination with IL-17A or IL-17F by IL-17-specific antibodies. **(A)** IL-17A (0.15 nM) or **(B)** IL-17F (25 nM) in combination with TNFα (0.025 nM) was pre-incubated with titrations of 496.g1, 496.g3 or an irrelevant antigen-specific human IgG1 isotype control for 1 h before addition to cells. NHDFs were subsequently stimulated for 18–20 h before IL-6 protein levels in the supernatant were determined by homogeneous time-resolved fluorescence. As shown in both figures, stimulation of NHDFs by TNF or IL-17A or IL-17F individually failed to elicit sufficient production of IL-6. Each condition was tested in duplicate and the data shown is representative of three separate experiments. Two-way ANOVA, Bonferroni post-test analysis was performed to compare inhibition of 496.g1 vs. 496.g3 (*p < 0.05, **p < 0.01, ***p < 0.001, ****p < 0.0001).

In order to determine whether the improvement in affinity for IL-17F had resulted in a concomitant improvement in neutralization activity, the potency of 496.g3 in neutralizing IL-17A- or IL-17F-stimulated release of IL-6 from NHDFs was compared with that of 496.g1. Stimulation of cells by IL-17A or IL-17F was too weak for a qualitative assay. Similarly, TNF only weakly stimulated cells. However, when either IL-17A or IL-17F was combined with TNF, they acted synergistically to strongly elevate the assay signal. Both 496.g1 and 496.g3 inhibited IL-17A and IL-17F to the level of stimulation seen with TNF alone (Figure 4). Pre-incubation of either antibody with TNF had no effect on IL-6 stimulation (data not shown). Equivalent IC_90_s were produced for 496.g1 and 496.g3 against IL-17A at 0.04 nM and 0.02 nM, respectively. Notably, 496.g3 was ~10-fold more potent than 496.g1 against IL-17F, with IC_90_ values of 23.41 nM and 238.8 nM, respectively.

To enable toxicology and pharmacokinetic studies in primates, the affinity of 496.g3 for cynomolgus macaque IL-17A and IL-17F was determined. 496.g3 showed similar affinity for human and cynomolgus macaque IL-17A, with K_D_ values of 3.2 pM and 12 pM, respectively (Table 2). While the affinity for cynomolgus macaque IL-17F at 345 pM was weaker than for human IL-17F at 23 pM (Table 2), it was considered to be sufficient for 496.g3 characterization in primate studies. Further studies showed 496.g3 did not bind to mouse or rat IL-17A or IL-17F (data not shown).

**Table 2:** Binding affinity and kinetic parameters of 496.g3 for human and cynomolgus macaque IL-17A and IL-17F.

**Species**	**Cytokine**	**k_**a**_ (M^**−1**^s^**−1**^)**	**k_**d**_ (s^**−1**^)**	**K_**D**_ (M)**	**K_**D**_ (pM)**
Human	IL-17F	2.99E+06	6.91E-05	2.31E-11	23
	IL-17A	4.59E+06	1.45E-05	3.17E-12	3.2
Cynomolgus macaque	IL-17F	4.27E+05	1.48E-04	3.45E-10	345
	IL-17A	1.64E+06	2.02E-05	1.23E-11	12

*Association (k_a_) and dissociation (k_d_) rates and affinity constants (K_D_) were determined by SPR*.

### Comparison With Approved Therapeutics

**Figure 5:**
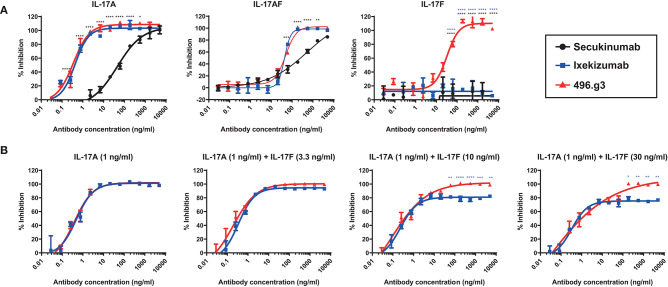
Comparative efficacy of 496.g3 with the approved anti-IL-17A antibodies secukinumab and ixekizumab in inhibiting IL-6 production by normal human dermal fibroblasts (NHDFs). As seen in panel **(A)** 496.g3 was the only antibody that inhibited IL-6 production in response to stimulation by all three cytokines. In panel **(B)**, 496.g3 maintained complete inhibition of IL-6 production, while the efficacy of ixekizumab declined with each stepwise increase in the ratio of IL-17F to IL-17A. Potency curves were calculated relative to NHDFs activated with TNF alone. In both sets of experiments, IL-17A (1 ng/mL), IL-17A/F (30 ng/mL), or IL-17F (30 ng/mL) in combination with TNF (1 ng/mL) were pre-incubated with titrations of 496.g3, ixekizumab **(A,B)** and secukinumab **(A)** for 1 h before addition to NHDFs. The cells were subsequently stimulated for 18–20 h before IL-6 protein levels in the supernatant were determined by homogeneous time-resolved fluorescence. Each condition was tested in duplicate and the data shown is representative of three separate experiments. Two-way ANOVA, Bonferroni post-test analysis was performed to compare inhibition of 496.g3 vs. secukinumab (black stars) or ixekizumab (blue stars) (* *p* < 0.05, ** *p* < 0.01, * *p* < 0.001, **** *p* < 0.0001).

To compare the efficacy of 496.g3 in an *in vitro* neutralization assay against anti-IL-17A-specific antibodies ixekizumab and secukinumab, the relative inhibition of IL-17A, IL-17F, and IL-17A/F signaling was examined. This assay utilized IL-6 release as a surrogate marker of inflammatory activation. The potency curves were calculated relative to fibroblasts activated with TNF alone. 496.g3 and ixekizumab showed similar IC_90_ values for IL-17A at 2.5 ng/mL and 2.2 ng/mL, respectively, and for IL-17A/F at 179.2 ng/mL and 91 ng/mL, respectively. In contrast, secukinumab was significantly less potent against IL-17A at 956.2 ng/mL. Of note, only 496.g3 demonstrated inhibition of IL-17F (IC_90_ 137.8 ng/mL), as neither ixekizumab nor secukinumab bound IL-17F (Figure 5A and Table 3). These data are consistent with the reported affinity constants for ixekizumab and secukinumab, and the measured affinities for 496.g3 (Table 4).

**Table 3:** Comparative activity of 496.g3 with anti-IL-17A-specific antibodies secukinumab and ixekizumab in neutralizing IL-17A, IL-17F an IL-17A/F in an *in vitro* neutralization assay.

	**IL-17A**		**IL-17A/F**		**IL-17F**	
	**IC_50_**	**IC_90_**	**IC_50_**	**IC_90_**	**IC_50_**	**IC_90_**
Secukinumab	45.0	956.2	525.9	65,535.0	ND	ND
Ixekizumab	0.4	2.2	43.2	91.0	ND	ND
496.g3	0.4	2.5	48.9	179.2	32.1	137.8

*Calculation of IC_50_ and IC_90_ values for 496.g3, secukinumab and ixekizumab were determined by four parameter non-linear regression analysis for IL-17A, IL-17A/F, and IL-17F. IC_50_ and IC_90_ values are measured in ng/ml. ND, Not detectable*.

**Table 4:** Binding affinities of 496.g3, secukinumab and ixekizumab for human IL-17A, IL-17A/F, and IL-17F.

**Cytokine**	**Secukinumab**	**Ixekizumab**	**496.g3**
IL-17A	129**^†^**	1.8^*^	3.2**^†^**
IL-17A/F	2,400^**^	1.8^*^	26**^†^^∧^**
IL-17F	NB**^†^**	NB^*^	23**^†^**

Internal

† and published

* *(31)*,

** *(32) binding affinities (pM) of 496.g3, secukinumab and ixekizumab for IL-17A, IL-17A/F, and IL-17F were generated by SPR. Affinity of 496.g3 for IL-17A and IL-17F was previously shown in Table 2*.

∧ *The kinetic parameters for binding to IL-17A/F are k_a_ = 3.19E+06, k_d_ = 8.17E-05 and K_D_ = 2.56E-11. NB, no binding*.

Measurements of IL-17A and IL-17F in psoriatic lesional tissue and serum show that, on average, the level of IL-17F is 30-fold higher than that of IL-17A (21). These quantitative differences are also observed in the serum of patients with spondyloarthritis (21). To determine whether the ratio of IL-17F to IL-17A in psoriatic lesions is important to the differential therapeutic potential of 496.g3 vs. ixekizumab *in vitro*, a neutralization assay was performed using varying ratios of IL-17F to IL-17A. In line with the previous experiment (Figure 5A), in the presence of IL-17A 1 ng/mL, 496.g3 and ixekizumab showed similar levels of inhibition. As the ratios of IL-17F to IL-17A were increased to 3:1, 10:1, and 30:1, 496.g3 maintained complete inhibition of IL-17-driven signaling (Figure 5B). In contrast, the efficacy of ixekizumab reduced with each stepwise increase in the ratio of IL-17F to IL-17A (Figure 5B). To achieve full inhibition at the increased IL-17F:IL-17A ratio, a concomitant increase in the concentration of 496.g3 was required; this was expected given the requirement to neutralize two ligands. Collectively, these data emphasize that neutralization of both IL-17A and IL-17F is required to fully suppress inflammation driven by these two structurally similar IL-17 cytokines.

## Discussion

Antibody 496.g3 (bimekizumab) was generated to test our hypothesis that both IL-17A and IL-17F contribute to chronic tissue inflammation, and that dual neutralization of IL-17A and IL-17F may lead to superior clinical outcomes compared with inhibition of IL-17A alone.

IL-17A is the principal therapeutic target of the IL-17 family, which may be reflected in the roles of IL-17A and IL-17F in mice. Studies using transgenic mice lacking either IL-17A or IL-17F showed that IL-17A plays a major role in the development of autoimmune diseases such as collagen-induced arthritis and Experimental Autoimmune Encephalitis (murine model of multiple sclerosis), and allergic diseases such as delayed-type hypersensitivity and contact hypersensitivity; in contrast, the role of IL-17F in these disease models was marginal (4, 5, 14, 33, 34). This is likely due to the 10,000-fold difference in potency between mouse recombinant IL-17A and IL-17F (data not shown). In contrast, the relative difference in potency between recombinant human IL-17A and IL-17F is ~100-fold (35).

**Figure 6:**
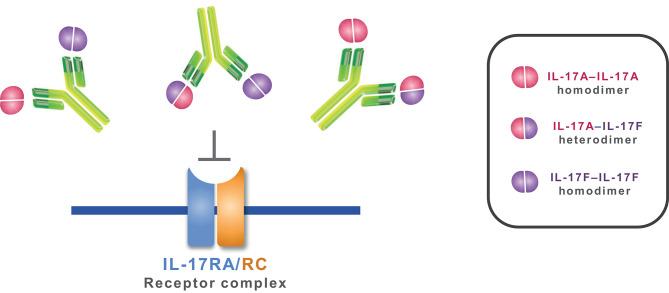
Mechanism of action of bimekizumab. By selectively binding to IL-17A, IL-17F, and the IL-17A/F heterodimer, bimekizumab inhibits the activation of the IL-17RA/RC receptor complex by these cytokines, and the subsequent inflammatory cascade.

In common with a number of other anti-IL-17A therapeutic antibodies that have entered the clinic, 496.g1, the antecedent of bimekizumab, was raised following immunization of rats with only IL-17A. 496.g1 possessed weak IL-17F neutralizing activity, which was subsequently exploited and enhanced through affinity maturation. As a standard IgG1, 496.g3 achieved dual targeting of both IL-17A and IL-17F (Figure 6). Biophysical characterization provided insights into how the molecule would behave in the manufacturing process. No unexpected or unwanted characteristics were identified during this process; indeed, 496.g3 showed good thermal stability and a favorable isoelectric point, which was comparable with other IgG monoclonal antibodies in the developmental and clinical landscape (36, 37).

Studies in human cells suggested a more prominent role for IL-17F in tissue inflammation (15). Treatment of synoviocytes from patients with rheumatoid arthritis with either recombinant IL-17A or IL-17F, in the presence of TNF, has been shown to induce a qualitatively similar gene signature (16). Transcriptional analysis of IL-17A- or IL-17F-stimulated dermal fibroblasts further reaffirmed the overlapping biology of these two cytokines, although IL-17A in either synoviocytes or dermal fibroblasts was the more potent cytokine (38). Indeed, using a more complex *in vitro* model of disease we have previously shown that dual neutralization of endogenous IL-17A and IL-17F produced by Th17 cells demonstrated greater suppression of inflammatory mediators compared to selective IL-17A blockade. Further, in addition to suppression of IL-6 we also observed significant inhibition of CXCL1, CXCL8, and CCL20, chemokines strongly linked to IL-17A and IL-17F biology (38). The limitations of our previous published research (38) with bimekizumab were two-fold. Firstly, 496.g3 was not compared against commercially approved anti-IL-17A antibodies, secukinumab, and ixekizumab, as performed in this study, but rather profiled against an in-house generated anti-IL-17A antibody. As our in-house anti-IL-17A antibody may behave differently to secukinumab and ixekizumab, it was more clinically relevant to compare against commercially approved antibodies. Further, in this study we also add more granularity on the specificity and potency of each antibody to specifically neutralize IL-17A, IL-17AF, or IL-17F when tested individually. Secondly, quantification of local and systemic levels of both IL-17 cytokines in patients with psoriasis, psoriatic arthritis, and ankylosing spondylitis revealed a greater abundance of IL-17F relative to IL-17A (>30-fold) (21). Significantly, we demonstrated, using an *in vitro* neutralization assay, that when the ratio of IL-17F to IL-17A was ≥10-fold the differential impact of 496.g3 over specific IL-17A inhibitors was observed. As expected, a higher concentration of 496.g3 was required to neutralize both IL-17A and IL-17F, when compared with IL-17A alone.

While the greater abundance of IL-17F and its shared overlapping biology with IL-17A suggest a role for this cytokine in promoting chronic tissue inflammation, our early clinical assessments of dual inhibition of IL-17A and IL-17F offers a direct approach to testing this hypothesis (39). Bimekizumab has completed Phase 1 and Phase 2 clinical trials in patients with psoriasis, psoriatic arthritis, and ankylosing spondylitis (38, 40–42). In the Phase 1 first-in-human, single-dose psoriasis study (NCT02529956), 26 patients received bimekizumab and 13 received placebo; bimekizumab treatment resulted in a rapid onset of clinically meaningful efficacy in measures of disease activity, which was maintained throughout the 20-week study in those receiving bimekizumab ≥160 mg (39). In the Phase 1b proof-of-concept study (NCT02141763) in patients with moderate to severe adult-onset psoriatic arthritis, 38 patients received bimekizumab and 12 patients received placebo (38). Bayesian analysis indicated a >99% probability that the American College of Rheumatology n (ACRn) index and ≥20% improvement in ACR index (ACR20) response at Week 8 were greater with bimekizumab vs. placebo and exceeded the pre-determined clinically relevant threshold. For the combined highest three doses, response rates at Week 8 were 80% (ACR20), 40% (ACR50), and 23% (ACR70), with maximal observed response rates for these endpoints of 80% (ACR20 at Week 8), 57% (ACR50 at Week 12), and 37% (ACR70 at Week 16). Moreover, in those patients with skin involvement, Week 8 Psoriasis Area Severity Index (PASI) response rates for PASI75 and PASI100 were 100% and 87%, respectively. Therefore, both pre-specified efficacy criteria in this study were met, demonstrating proof-of-concept. Further to this, results from BE ABLE 1, a 12-week, randomized, double-blind, placebo-controlled Phase 2b study in patients with moderate to severe psoriasis demonstrated superior efficacy of bimekizumab vs. placebo in all primary and secondary endpoints (40). Significant dose-dependent responses were observed and, in the highest dose groups, patients achieved high levels of skin clearance (PASI90) at Week 12. Of note, ~50–60% of patients in the three highest dose groups achieved complete skin clearance (PASI100) following 12 weeks of bimekizumab treatment. Importantly, in all three clinical studies, the safety findings observed were consistent across bimekizumab dose groups and were as expected when considered in the context of anti-IL-17A antibodies. Preclinical and early clinical data are encouraging and further support the targeted approach of dual neutralization of IL-17A and IL-17F.

Results of the studies reported here demonstrate that bimekizumab, a monoclonal antibody, potently and selectively neutralizes IL-17A and IL-17F. With promising early clinical data in psoriasis, psoriatic arthritis, and ankylosing spondylitis, dual inhibition of IL-17A and IL-17F with bimekizumab offers a new therapeutic approach for the treatment of patients with immune-mediated inflammatory diseases.

## Data Availability Statement

Data from non-clinical studies is outside of UCB's data sharing policy and is unavailable for sharing.

## Author Contributions

RA, AM, TB, AL, RP, SR, SW, and MG were involved in antibody development and characterization, analyzed, and interpreted the data. RO and PV provided mathematical modeling. AM, AL, and SS provided conceptual and supervisory support. RA, AM, and MG drafted the manuscript. All authors contributed revisions, approved the final manuscript, contributed to the article, and approved the submitted version.

## Conflict of Interest

RA, AM, TB, AL, RO, RP, SR, SS, PV, and MG are employees of UCB Pharma and hold stocks and/or stock options in UCB Pharma. SW is an employee of UCB Pharma.
