# Supplementary material for: Bimekizumab, a Novel Humanized IgG1 Antibody That Neutralizes Both IL-17A and IL-17F
Source: Front Immunol. 2020 Aug 21;11:1894. doi: 10.3389/fimmu.2020.01894 (PMC7473305; doi:10.3389/fimmu.2020.01894)
Supplement: Supplementary file 1 [file Data_Sheet_1.pdf]

SUPPLEMENTARY MATERIAL

496.g1 light chain variable region

AIQLTQSPSSLSASVGDRVTITCRADESVTTLMHWYQQKPGKAPKLLIYLVSNRESGVPSRFSGS
GSGTDFTLTISSLQPEDFATYYCQQTWSDPWTFGQGTKVEIK

496.g3 light chain variable region – 5 mutations are underlined

AIQLTQSPSSLSASVGDRVTITCRADESVRTLMHWYQQKPGKAPKLLIYLVSNSEIGVPDRFSGS
GSGTDFRLTISSLQPEDFATYYCQQTWSDPWTFGQGTKVEIK

496.g1 / 496.g3 heavy chain variable region

EVQLVESGGGLVQPGGSLRLSCAASGFTFSDYNMAWVRQAPGKGLEWVATITYEGRNTYYRD
SVKGRFTISRDNAKNSLYLQMNSLRAEDTAVYYCASPPQYYEGSIYRLWFAHWGQGTLVTVSS
